# Development and efficacy evaluation of a competency-based training program for pediatric operating room nurses

**DOI:** 10.3389/fmed.2026.1769565

**Published:** 2026-03-06

**Authors:** Qingqing Du, Zhen Wang, Mengfan Xia, Jing Cao, Yingmin Liu, Ziwei Jin, Xia Yang

**Affiliations:** 1 Shanghai Children’s Hospital, School of Medicine, Shanghai Jiao Tong University, Shanghai, China; 2 Tongji University School of Medicine, Shanghai, China

**Keywords:** effectiveness evaluation, job competency, nurse training, nursing education, pediatric operating theater, program development

## Abstract

**Background:**

The complex and high-stakes environment of the pediatric operating room (OR) demands nurses with exceptional competency. Traditional training models often fall short in preparing new nurses for these unique challenges. This study aimed to develop and evaluate the effectiveness of a structured competency-based training (CBT) program for newly recruited pediatric OR nurses.

**Methods:**

A two-round Delphi method involving 15 experts was used to develop the CBT program framework. A prospective, single-center pilot study with a quasi-experimental design was conducted with 40 newly recruited nurses from a tertiary pediatric hospital in Shanghai. Participants were randomly assigned using a computer-generated random number sequence and sealed opaque envelopes to either the experimental group (*n* = 20), which received the 8-week CBT program incorporating scenario simulation, case-based learning, and workshops, or the control group (*n* = 20), which received conventional one-to-one mentoring. Outcome assessors were blinded to group allocation. Outcomes were assessed pre- and post-intervention using theoretical tests, practical skill evaluations, the Operating Room Nurse Competency Scale, the General Self-Efficacy Scale (GSES), and the Perceived Ability-Job Fit Scale.

**Results:**

Post-training, the experimental group showed significantly greater improvements than the control group across all measured outcomes. Specifically, the experimental group achieved higher scores in theoretical knowledge (88.52 ± 4.54 vs. 80.23 ± 6.31, *p* < 0.001), practical skills (85.22 ± 4.83 vs. 78.36 ± 5.61, *p* < 0.001), overall job competency (92.49 ± 4.72 vs. 82.72 ± 5.28, p < 0.001), self-efficacy (26.52 ± 4.61 vs. 21.42 ± 5.87, *p* < 0.01), and perceived ability-job fit (18.32 ± 2.98 vs. 14.52 ± 3.03, *p* < 0.001).

**Conclusion:**

The systematic, competency-based training program developed in this study proved to be more effective than traditional mentoring in enhancing the knowledge, skills, professional competency, self-efficacy, and role adaptation of new pediatric OR nurses. This model offers an evidence-based and practical framework for optimizing training and supporting the professional development of nurses in specialized pediatric surgical settings.

## 1 Introduction

Pediatric operating room nursing represents a highly specialized domain within clinical care, characterized by the vulnerability of the patient population, rapid disease progression, and the technical complexity of surgical interventions. Nurses in this setting are required to possess not only meticulous operational skills but also exceptional abilities in clinical observation, rapid assessment, and emergency response (1). Compared to adult care, pediatric practice places greater emphasis on developmental considerations, family-centered communication, and the management of heightened physiological and emotional sensitivities, thereby imposing significantly elevated professional demands.

This study is grounded in two established theoretical frameworks: Bandura’s Self-Efficacy Theory and the Kirkpatrick Evaluation Model. According to Bandura (2), self-efficacy—defined as an individual’s belief in their capability to execute behaviors necessary to produce specific performance attainments—is developed through four primary sources: performance accomplishments (mastery experiences), vicarious experiences, verbal persuasion, and emotional arousal. Competency-based training enhances self-efficacy by providing structured mastery experiences through scenario simulation and deliberate practice, thereby facilitating the transition from novice to competent practitioner (3).

For program evaluation, we employed the Kirkpatrick Model, which assesses training effectiveness at four levels: Reaction (trainee satisfaction), Learning (knowledge and skills acquisition), Behavior (transfer of learning to practice), and Results (organizational impact) (4). This framework ensures comprehensive evaluation beyond immediate learning outcomes to encompass behavioral change and professional competence development (5). These theoretical foundations directly informed the design of our competency-based training program and the selection of outcome measures.

Within conventional training frameworks, newly recruited nurses in pediatric operating rooms often experience limited opportunities for proactive learning and exhibit low engagement in educational activities. This frequently results in suboptimal training outcomes, hindering the achievement of clinical instructional goals and delaying the nurse’s capacity to perform independently. In response to these challenges, the National Nursing Development Plan (2021–2025) explicitly advocates for the establishment of a competency-oriented training system, emphasizing role-specific, categorized, and stratified approaches to enhance the clinical efficacy of nursing staff (6). In this context, job competency—defined as the integrated manifestation of knowledge, skills, and professional attitude in clinical practice—directly influences nursing quality and patient safety.

Although core competency cultivation in nursing has been widely explored both domestically and internationally, systematic training programs tailored to the distinctive environment of the pediatric operating room remain scarce. There is a particular lack of empirical studies that integrate knowledge, skills, personal attributes, and motivation into a cohesive educational framework. Recent evidence suggests that active learning strategies—such as scenario simulation and case-based teaching—can significantly enhance clinical decision-making and emergency response abilities among specialized nurses (7), offering promising directions for pediatric operating room training.

This pilot study aims to address these gaps by developing, implementing, and preliminarily evaluating a competency-based training program specifically designed for pediatric OR nurses. Using a randomized controlled design, we compared its effectiveness against traditional one-to-one mentoring. We hypothesized that the structured, competency-driven approach would lead to superior outcomes in theoretical knowledge, practical skills, professional competency, self-efficacy, and perceived job fit at the Learning level of Kirkpatrick’s model, with potential implications for behavioral change.

## 2 Subjects and methods

### 2.1 Study design and population

This prospective, single-center, quasi-experimental pilot study was conducted at a tertiary pediatric hospital in Shanghai between August 2023 and August 2025. Forty newly recruited nurses rotating through the pediatric operating theater were selected using convenience sampling. No statistically significant differences were observed between the two groups in baseline characteristics, including age, gender, and educational attainment (*p* > 0.05), rendering them comparable.

#### 2.1.1 Sample size justification

As a pilot feasibility study, the sample size was determined based on practical considerations and established guidelines for pilot studies in nursing education. The National Center for Complementary and Integrative Health (NCCIH) recommends that pilot studies include sufficient participants to evaluate feasibility outcomes such as recruitment rates, retention, and intervention fidelity, typically 15–30 per group for quantitative studies (8). With 20 participants per group, this study provides adequate power to detect large effect sizes (Cohen’s *d* ≥ 0.8) with 80% power at *α* = 0.05, consistent with pilot study objectives of assessing preliminary efficacy and informing future full-scale trials.

#### 2.1.2 Inclusion criteria

① Years of service ≤2 years; ② Possession of a valid nursing license; ③ Voluntary signing of informed consent to participate.

#### 2.1.3 Exclusion criteria

① Nursing staff in training, internship, or probationary periods; ② Absence during the study period; ③ Failure to complete standardized pre-service training.

#### 2.1.4 Randomization and allocation concealment

Participants were randomly assigned to experimental and control groups using a computer-generated random number sequence (SPSS 27.0) with a 1:1 allocation ratio. The randomization sequence was generated by an independent statistician not involved in participant recruitment or assessment. Group assignments were placed in sequentially numbered, opaque, sealed envelopes to ensure allocation concealment (9). Envelopes were opened sequentially only after participants completed baseline assessments and written informed consent.

#### 2.1.5 Blinding

Due to the nature of the educational intervention, participants and trainers could not be blinded to group allocation. However, outcome assessors (research assistants responsible for data collection and entry) were blinded to group assignment to minimize detection bias (10).

### 2.2 Development of the training program

#### 2.2.1 Theoretical basis

The training program was designed based on three complementary frameworks: Miller’s Pyramid of Clinical Competence, Bandura’s Self-Efficacy Theory, and the Kirkpatrick Evaluation Model.

Miller’s Pyramid provides a hierarchical framework for competency assessment spanning from “Knows” (knowledge) to “Knows How” (application), “Shows How” (simulated practice), and “Does” (real-world performance) (11). This framework guided the progressive structure of our curriculum, ensuring alignment between educational activities and competency levels.

Bandura’s Self-Efficacy Theory informed the pedagogical strategies employed in the program. By emphasizing mastery experiences through repeated scenario simulation and deliberate practice, the program was designed to enhance participants’ confidence in their clinical capabilities (2, 3).

The Kirkpatrick Evaluation Model structured our outcome assessment approach, focusing on Level 2 (Learning) outcomes in this pilot study, with the framework allowing for future expansion to behavioral and results-level evaluation (4, 5).

#### 2.2.2 Expert selection criteria

Focusing on comprehensive representation across the entire pediatric operating theater nursing chain, the 15 experts selected must concurrently meet the following criteria: ① Domain coverage encompassing nursing management (pediatric operating theater head nurses/departmental head nurses), clinical nursing (specialist pediatric operating theater nurses with over 10 years’ experience), and nursing education (pediatric nursing faculty members/clinical preceptors from nursing institutions); ② Hold an associate senior or higher professional title; ③ Have participated in pediatric surgical nursing training, research, or guideline development within the past 5 years, with demonstrable academic output. The panel size of 15 experts aligns with Delphi methodology recommendations of 12–15 experts for reliable consensus achievement (12).

#### 2.2.3 Delphi process and item generation

The Delphi process followed established guidelines for modified Delphi studies in nursing research (13). In Round 1, an initial item pool was generated through comprehensive literature review, analysis of national competency standards for operating room nurses, and semi-structured interviews with 5 experienced pediatric OR nurses and 3 nurse managers. These qualitative data were analyzed using directed content analysis to identify key competency domains, yielding 38 preliminary items across four proposed modules.

The Round 1 questionnaire included open-ended questions allowing experts to propose additional items, comment on item clarity, and suggest modifications. Qualitative feedback was synthesized thematically and used to refine item wording, eliminate redundancy, and add 4 new items proposed by experts. In Round 2, experts rated the importance (1–5 Likert scale) and feasibility of each item. Consensus was determined using the dual-criteria approach: mean importance score ≥4.0 and coefficient of variation <0.25. Inter-rater agreement was assessed using Kendall’s coefficient of concordance (*W*).

##### 2.2.3.1 Results indicated

Expert authority coefficient [Cr = (judgment basis + familiarity)/2] reached 0.87; 100% valid questionnaire return rate across two rounds. First-round *W* = 0.62 (*p* < 0.05), second-round *W* increased to 0.78 (*p* < 0.01), with all items meeting dual-criterion requirements.

The iterative Delphi process ensured content validity through expert consensus and qualitative feedback integration, resulting in a comprehensive, contextually appropriate competency framework, resulting in a comprehensive, contextually appropriate competency framework.

#### 2.2.4 Outcomes of program development

The final framework comprises four core modules: specialized knowledge, professional competence, personal attributes, and individual motivation. These cover targeted content, including pediatric surgical assistance procedures, perioperative care, infection prevention and control, emergency scenario management, nurse–patient communication and team collaboration, professional ethics, and psychological resilience.

### 2.3 Training implementation

The control group employed a traditional one-to-one mentoring approach with an 8-week training cycle. The experimental group utilized competency-based training structured around the aforementioned four modules, incorporating diverse teaching methods.

#### 2.3.1 Detailed curriculum structure

The 8-week CBT program consisted of 96 total academic hours (12 h/week), allocated as follows.

Theoretical instruction (32 h): 4 h weekly covering pediatric surgical anatomy, pathophysiology, and evidence-based perioperative care.
Practical skills training (48 h): 6 h weekly in a simulation center, focusing on instrument handling, sterile technique, and emergency procedures.
Scenario simulation (16 h): Bi-weekly 4-h sessions involving high-fidelity simulation of pediatric perioperative emergencies.
Specific scenario simulation examples: Severe pediatric hemorrhage—management of massive transfusion protocol activation, hemorrhagic shock recognition, and team coordination.
Case-based learning (CBL): Weekly 2-h sessions using authentic pediatric surgical cases (e.g., laparoscopic appendectomy) to develop clinical reasoning and decision-making skills.
Remediation policy: To ensure competency-based progression, a structured remediation policy was implemented. Participants scoring below 80% on weekly formative assessments (both theoretical and practical) were required to receive mandatory remedial instruction and practice sessions. Remediation involved one-on-one tutoring with specialist nurses, additional simulation sessions targeting identified weakness areas, and re-assessment within 48 h. Trainees progressed to subsequent modules only after demonstrating satisfactory competency (≥80% score). This approach ensured that no participant proceeded with significant knowledge or skill gaps, embodying the principle that time is variable while standards remain constant.
The teaching team comprised: One Deputy Senior or above Pediatric Operating Theater Nurse Manager overseeing the program; two specialist nurses with over 10 years’ experience delivering scenario simulations and case studies; and one Nursing Education Mentor responsible for personal trait guidance.

### 2.4 Evaluation criteria

#### 2.4.1 Theoretical examination results

The theoretical examination consisted of a structured 100-item multiple-choice test developed by the research team in consultation with pediatric surgical nursing experts. The examination covered three main domains: pediatric surgical nursing knowledge (50 items, 50%), infection control principles (30 items, 30%), and emergency care protocols (20 items, 20%). Questions were categorized by cognitive level according to Bloom’s taxonomy: 40% knowledge recall, 35% application, and 25% analysis and problem-solving. Each correct answer scored 1 point, with no partial credit. The maximum possible score was 100 points.

Assessment criteria scores ≥90: Excellent; 80–89: Good; <80: Requires remediation. The examination was administered in a proctored classroom setting with a 90-min time limit. To ensure content validity, the test blueprint was developed based on the competency framework established through the Delphi process, and questions were reviewed by an independent panel of three pediatric OR nurse specialists for relevance and clarity.

#### 2.4.2 Practical examination results

Practical skills were assessed using Objective Structured Clinical Examinations (OSCEs) aligned with Miller’s “Shows How” level (11). Two standardized pediatric surgical scenarios were selected based on the following criteria: (1) frequency of occurrence in pediatric surgical practice, (2) representation of distinct complexity levels, and (3) comprehensive assessment of core competencies required for pediatric OR nursing.

Specifically, we selected:

General surgery scenario: Inguinal hernia repair—chosen as one of the most common pediatric surgical procedures globally, allowing assessment of fundamental perioperative nursing skills including patient positioning, sterile technique, and routine instrument handling.
Specialized surgery scenario: Neonatal laparotomy—selected to represent high-complexity, high-acuity surgical care, thermal regulation, and coordination with multidisciplinary teams.

The OSCE examination comprised four sequential stations, each lasting 15 min with 5-min transition intervals: (1) Preoperative preparation and patient assessment, (2) Sterile field establishment and instrument management, (3) Intraoperative assistance and emergency response, and (4) Postoperative handover and documentation. Each station utilized standardized patients (pediatric manikins) and standardized checklists derived from the Operating Room Nurse Competency Scale domains.

The structured checklists contained 25 specific items across five skill categories: technical competencies (sterile technique, 5 items; instrument handling, 5 items), clinical decision-making (4 items), emergency response (5 items), teamwork and communication (3 items), and professional attitude (3 items). Each item was rated on a 4-point Likert scale (0 = not performed, 1 = performed incorrectly, 2 = performed with prompting, 3 = performed independently and correctly), yielding a maximum score of 75 points per scenario. Raw scores were converted to percentage scores for analysis.

Two independent assessors evaluated performance using structured checklists. The checklists included technical competencies (sterile technique, instrument handling), emergency response, and teamwork (Cronbach’s *α* = 0.89). Inter-rater reliability between assessors was 0.85 (ICC), indicating good agreement.

Additionally, to provide granular insight into learning gaps, we conducted item-level analysis of specific skill domains. The assessment rubrics explicitly evaluated: (a) emergency response skills (recognition of deteriorating vital signs, activation of hemorrhage protocols, calculation of pediatric drug dosages), (b) complex instrument handling (laparoscopic equipment assembly, microsurgical instrument preparation), (c) developmental care (temperature maintenance for neonates, age-appropriate positioning), and (d) communication (family interaction, surgical team coordination). Performance in each domain was recorded separately to identify specific areas of strength and weakness.

### 2.5 Evaluation criteria

#### 2.5.1 Theoretical examination results

A monthly theoretical assessment is conducted, covering pediatric surgical nursing knowledge, infection control, and emergency care. The examination is scored out of 100 points to evaluate nursing staff’s mastery of professional theory.

#### 2.5.2 Practical examination results

This study establishes two categories of practical assessment: general surgery and specialized surgery. Assessments are conducted on-site by education nurses and head nurses, focusing on evaluating procedural compliance, emergency response capabilities, and teamwork proficiency.

#### 2.5.3 Operating theater nurse competency evaluation

This study employs the Operating Theater Nurse Competency Evaluation Scale developed by Abusubhiah et al. (3). This scale comprises four dimensions: specialized knowledge, professional competence, personal traits, and motivational factors, totaling 24 items. A five-point Likert scale was employed, with scores of 24–56 indicating low competency, 57–88 moderate competency, and 89–120 high competency. Higher scores denote stronger job competency. Reliability coefficients for each dimension ranged from 0.850 to 0.967.

#### 2.5.4 General self-efficacy

The General Self-Efficacy Scale (GSES) was employed. This instrument was originally developed by German scholars, including Schwarzer, subsequently translated and adapted into Chinese by Wang et al. (14). The scale comprises 10 items covering confidence in handling unexpected events, effort invested in problem-solving, ability to maintain composure under pressure, and trust in one’s own coping capabilities. Employing a 4-point Likert scale, the total score ranges from 10 to 40 points. Scores of 10–13 are classified as low, 14–27 as moderate, and 28–40 as high, with higher scores indicating stronger self-efficacy. The scale demonstrated internal consistency reliability (Cronbach’s *α*) of 0.824.

#### 2.5.5 Perceived ability-job fit

This study employed the Perceived Ability-Job Fit Scale developed by Abdel-Halim and revised by Fields (15), designed to assess individuals’ subjective perceptions of their ability-job compatibility. The scale comprises five items covering dimensions such as whether personal abilities are fully utilized at work, whether one possesses the necessary competencies for the role, whether one undertakes tasks best suited to their strengths, and whether one feels adequately prepared for the job. Scoring employs a five-point Likert scale, yielding total scores ranging from 5 to 25 points. Scores of 5–11 indicate low levels, 12–18 denote moderate levels, and 19–25 signify high levels, with increasing scores reflecting stronger subjective perceptions of ability-job fit. The scale demonstrates internal consistency reliability with a Cronbach’s *α* coefficient of 0.840.

### 2.6 Statistical methods

Data analysis was performed using SPSS 27.0 software. Quantitative data are presented as mean ± standard deviation ( $\bar{x}\pm s$ ). Following verification of normality and homogeneity of variance, independent samples *t*-tests were employed to compare differences between groups. Item-level analysis of practical skills was conducted using descriptive statistics to identify specific learning gaps. Effect sizes (Cohen’s *d*) were calculated for all significant findings to assess practical significance. Differences were considered statistically significant at *p* < 0.05.

## 3 Results

### 3.1 Comparison of theoretical and practical performance

Following the training intervention, nurses in the experimental group, who received the competency-based program, achieved significantly higher scores in both theoretical knowledge and practical skills compared to those in the control group who underwent traditional mentoring (all *p* < 0.001). The detailed results are presented in Table 1.

**Table 1:** Comparison of theoretical and practical examination scores between the two groups (mean ± SD).

Group	Number	Theoretical score	Practical score
Control group	20	80.23 ± 6.31	78.36 ± 5.61
Experimental group	20	88.52 ± 4.54	85.22 ± 4.83
*t*-value		4.77	4.14
*p*-value		<0.001	<0.001

### 3.2 Detailed analysis of practical skill domains

Item-level analysis of the OSCE results revealed specific patterns of performance across skill domains. While the experimental group outperformed the control group in all domains (*p* < 0.05), both groups demonstrated relatively lower mean scores in emergency response scenarios and complex instrument handling compared to routine sterile technique and patient positioning. Notably, the experimental group showed significantly smaller performance decrements in high-stress emergency scenarios compared to the control group (effect size *d* = 1.34), suggesting enhanced readiness for critical situations (Table 2).

**Table 2:** Comparison of specific skill domain performance between groups (mean ± SD).

Skill domain	Experimental group (*n* = 20)	Control group (*n* = 20)	*t*-value	*p*-value
Emergency response	82.5 ± 5.2	74.3 ± 6.8	4.21	<0.001
Complex instrument handling	84.1 ± 4.9	76.2 ± 5.4	4.89	<0.001
Routine sterile technique	88.3 ± 3.1	81.4 ± 4.2	5.92	<0.001
Patient positioning	87.8 ± 3.5	80.9 ± 4.8	5.14	<0.001

#### 3.2.1 Remediation outcomes

Four participants in the experimental group (20%) and 11 in the control group (55%) required remediation after initial practical assessments. All remediated participants subsequently achieved competency standards (≥80%) upon re-testing.

### 3.3 Comparison of job competency scores

Analysis of the job competency scale revealed that the experimental group scored significantly higher than the control group across all four dimensions—specialized knowledge, professional competence, personal traits, and motivation—as well as in the total score (all *p* < 0.05). The comparative data are shown in Table 3.

**Table 3:** Comparison of job competency scores between the two groups (mean ± SD).

Group	Number	Specialized knowledge	Professional competence	Personal traits	Motivation	Total score
Control group	20	29.25 ± 3.22	16.47 ± 3.12	24.55 ± 1.55	12.45 ± 2.31	82.72 ± 5.28
Experimental group	20	32.51 ± 2.46	18.56 ± 2.84	26.41 ± 2.11	15.01 ± 1.92	92.49 ± 4.72
*t*-value		3.60	2.35	3.44	3.75	6.17
*p*-value		<0.001	<0.05	<0.01	<0.001	<0.001

### 3.4 Comparison of self-efficacy scores

A significant between-group difference was also observed in self-efficacy. The mean self-efficacy score in the experimental group was markedly higher than that in the control group (*p* < 0.01). This finding aligns with Bandura’s theory that structured mastery experiences through simulation and deliberate practice enhance self-efficacy beliefs (2). The results are presented in Table 4.

**Table 4:** Comparison of self-efficacy scores between the two groups (mean ± SD).

Group	Number	Self-efficacy score
Control group	20	21.42 ± 5.87
Experimental group	20	26.52 ± 4.61
*t*-value		3.01
*p*-value		<0.01

### 3.5 Comparison of competency-job fit scores

Regarding competency-job fit scores, the experimental group (18.32 ± 2.98) significantly exceeded the control group (14.52 ± 3.03), with a statistically significant difference (*p* < 0.05), as shown in Table 5.

**Table 5:** Comparison of competency-job fit scores between the two groups (mean ± SD).

Group	Number	Competence-job fit score
Control group	20	14.52 ± 3.03
Experimental group	20	18.32 ± 2.98
*t*-value		4.08
*p*-value		<0.001

## 4 Discussion

### 4.1 Theoretical contribution and interpretation

The findings of this study demonstrate that a structured competency-based training program yields superior outcomes compared to traditional one-to-one mentoring for newly recruited pediatric operating room nurses. While the research was designed as a pragmatic educational intervention, the results resonate with established learning theories, particularly experiential learning and competency-based education principles (7, 16).

The marked improvement in self-efficacy scores among experimental group participants suggests that the CBT program successfully created mastery experiences through repeated practice in simulated environments. This aligns with the understanding that confidence in professional capabilities develops not merely from observation but from concrete experiences where learners can test their skills, receive feedback, and refine their performance (1, 7). The cyclical nature of the training—alternating between theoretical instruction, simulation practice, and case analysis—likely facilitated deeper cognitive processing and skill retention than the linear apprenticeship model of traditional mentoring.

Viewing the results through the lens of competency-based medical education highlights a fundamental shift from time-based training to outcome-based learning. Rather than assuming competence after a fixed duration, the CBT program required demonstrable proficiency across four distinct dimensions before completion (6, 17). This approach is particularly salient in pediatric surgical settings where technical errors can have severe consequences, ensuring that nurses are truly prepared for independent practice rather than merely credentialed by attendance (18).

The substantial gains in perceived ability-job fit merit particular attention. New graduates typically experience a gap between their educational preparation and the complex demands of professional practice, often described as transition shock (19, 20). The structured nature of the CBT program—providing clear expectations, progressive skill development, and explicit competency standards—appears to have bridged this gap more effectively than the variable quality often associated with informal mentoring relationships (21). Trainees could visualize their progression through defined competency milestones, likely enhancing their sense of professional identity and role clarity.

The item-level analysis revealing relative weaknesses in emergency response and complex instrument handling, even within the high-performing experimental group, suggests that certain high-acuity, low-frequency skills may require extended deliberate practice beyond the current 8-week curriculum. These specific learning gaps highlight the importance of continuous curriculum refinement based on granular performance data.

### 4.2 Understanding the mechanisms of change

The superior performance of the experimental group likely stems from the synergistic interaction of multiple instructional strategies rather than any single element. Scenario simulation created psychological safety for encountering high-acuity, low-frequency events—such as severe pediatric hemorrhage—that trainees might otherwise encounter only after months or years of practice (1). In these simulated scenarios, errors became learning opportunities rather than patient safety events, allowing for the repetition and refinement essential to skill mastery.

Case-based learning complemented simulation by developing the clinical reasoning necessary for operating room nursing. Through analysis of authentic pediatric surgical cases, trainees learned to integrate anatomical knowledge, physiological principles, and procedural considerations into coherent clinical decisions (16). This active cognitive engagement contrasts with the passive observation that characterizes much traditional mentoring, where novices may spend considerable time watching without participating in the analytical work of patient care.

The remediation component of the program represented another critical difference from conventional training. Our finding that 20% of experimental group participants required remediation (compared to 55% in the control group), with all achieving competency upon re-testing, validates the effectiveness of the structured remediation policy. This mandatory remediation—involving targeted one-on-one tutoring and additional simulation sessions—ensured that no trainee progressed with significant skill deficits, embodying the CBME principle that time should be variable while standards remain constant (17). In traditional models, struggling trainees might progress through the rotation without addressing competency gaps, leaving deficiencies to be discovered later in independent practice. The CBT program’s mandatory remediation ensured that all participants achieved minimum standards before moving forward.

Beyond technical skills, the explicit attention to personal attributes and motivation addressed dimensions often neglected in clinical training. Pediatric operating room nursing demands not only manual dexterity and clinical knowledge but also emotional regulation, ethical reasoning, and effective communication with anxious families (19, 21). By incorporating these elements into the formal curriculum rather than assuming they would develop organically, the program acknowledged the complex nature of professional competence in high-stakes surgical environments.

### 4.3 Relationship to existing evidence

These findings corroborate an emerging body of literature supporting structured, active learning approaches in nursing education. Zhang et al. (1) demonstrated that problem-based learning combined with scenario simulation improved critical thinking and decision-making among operating room nurses, consistent with our observations of enhanced practical skills and professional competence. Similarly, Wang and Lin’s (16) implementation of a PDCA scenario-based teaching model for junior pediatric nurses yielded improvements in core competencies that parallel our findings.

The self-efficacy results warrant particular consideration given their implications for retention and professional development. Previous research has documented the erosion of confidence that often accompanies the transition from student to professional nurse, with some studies linking low self-efficacy to intentions to leave the profession (19, 20). The significant improvement in GSES scores among CBT participants suggests that structured training can interrupt this trajectory, potentially supporting both individual career sustainability and organizational workforce stability.

Our findings regarding perceived ability-job fit also connect with broader discussions of transition shock in nursing literature. Zhu et al. (21) demonstrated that transition shock negatively impacts patient safety competence, with professional spirit serving a moderating role. The CBT program’s explicit focus on psychological resilience and professional identity may have functioned similarly, buffering trainees against the disorientation often experienced during early professional practice. The significantly higher ability-job fit scores suggest that participants felt genuinely prepared for their responsibilities rather than merely surviving their orientation period (22).

### 4.4 Limitations and future directions

Several limitations temper the conclusions that can be drawn from this study. The single-center design at a tertiary pediatric hospital with established simulation facilities and specialized faculty means that the findings may not transfer directly to institutions with different resource levels or patient populations. The competencies prioritized in our Delphi process—while reflecting expert consensus—may require adaptation for other pediatric surgical contexts.

The sample size, while adequate for detecting the large effects observed in this pilot study, precludes detection of smaller but potentially meaningful differences and prohibits subgroup analyses. We cannot determine whether the CBT program was equally effective for trainees with different educational backgrounds or baseline competency levels. The absence of long-term follow-up means that we cannot assess whether the observed gains persist over time or whether they translate into sustained improvements in clinical practice and patient outcomes.

The inability to blind participants and trainers to group assignment introduces the possibility of performance bias. Enthusiasm for the novel CBT program may have motivated experimental group participants to exert greater effort, while control group participants might have experienced disappointment that influenced their engagement or assessment performance. Future investigations might employ cluster randomization or wait-list designs to mitigate these concerns.

Finally, the immediate post-training assessment captures only short-term learning outcomes. As outlined in the Kirkpatrick Evaluation Model, training effects should ultimately be evaluated at the behavioral and results levels—changes in workplace practice and organizational outcomes. Our study assessed only the learning level, leaving questions about real-world application unanswered. The specific learning gaps identified in emergency response and complex instrument handling suggest that future studies should include longitudinal tracking to determine whether these initial weaknesses translate into persistent competency deficits or whether they resolve with clinical experience.

Despite these limitations, the findings suggest that structured CBT offers a viable alternative to traditional mentoring. Institutions implementing similar programs should establish clear competency standards, ensure adequate simulation resources, and mandate remediation for trainees not meeting benchmarks.

Future studies should employ multi-center designs with larger samples and longer follow-up periods to confirm these preliminary findings and assess long-term impact on nursing practice and patient outcomes. Additionally, longitudinal studies tracking participants’ career development and retention rates would provide valuable insights into the sustained benefits of competency-based training.

## 5 Conclusion

This study has certain limitations: as a pilot study, the sample size was small and it was a single-center investigation; training effectiveness was primarily evaluated through immediate outcomes, lacking mid-to-long-term follow-up data to observe the durability of effects. Despite these limitations, the large effect sizes and consistent improvements across all measured outcomes support the feasibility and preliminary efficacy of the CBT program. Future research could expand the sample size, conduct multi-center validation, and undertake longitudinal studies to assess the long-term impact of this training program on nurses’ career development, nursing quality, and pediatric patient outcomes. Additionally, exploring the integration of this model with digital learning tools could address some resource and scalability challenges.

## Data Availability

The raw data supporting the conclusions of this article will be made available by the authors, without undue reservation.
